# Formation of sclerotia in * Sclerotinia ginseng* and composition of the sclerotial exudate

**DOI:** 10.7717/peerj.6009

**Published:** 2018-11-23

**Authors:** Dan Wang, Junfan Fu, Rujun Zhou, Zibo Li, Yujiao Xie, Xinran Liu, Yueling Han

**Affiliations:** Department of Plant Protection, Shenyang Agricultural University, Shenyang, Liaoning, China

**Keywords:** Metabolite composition, Sclerotia, Natural products, Exudate droplets, Sclerotinia ginseng

## Abstract

**Background:**

*Sclerotinia ginseng* is a major devastating soil-borne pathogen of ginseng that can cause irreparable damage and large economic losses. This pathogen produces sclerotia, which are among the most persistent resting structures produced by filamentous fungi. The production of an exudate is a common feature of sclerotial development.

**Methods:**

*S. ginseng* was cultured on 10 different media and the following parameters were measured: mycelial growth rate (mm/day), initial formation time of exudate droplets, total quantity of exudate, number of sclerotia per dish, and sclerotial fresh/dry weight. The composition of the sclerotial exudate was analyzed using four methods (high performance liquid chromatography, gas chromatography-mass spectrometry, flame atomic absorption spectrometry, and Nessler’s reagent spectrophotometry).

**Results:**

We found that PDA was the optimal medium for exudate production, while SDA medium resulted in the highest mycelial growth rate. The earliest emergence of exudate droplets from sclerotia was on OA-YE and V8 media. The largest amount of sclerotia and the smallest sclerotia were produced on V8 medium. The maximum and minimum dry/fresh weight were obtained on MEA medium and V8 medium, respectively. The exudate contained organic acids (oxalic acid, gallic acid, ferulic acid, vanillic acid, caffeic acid, and tannic acid), carbohydrates (inositol, glucose, and trehalose), various ions (potassium, sodium, and magnesium), and ammonia.

**Discussion:**

The functions of the identified compounds are discussed within the context of pathogenicity, sclerotial development, and antimicrobial activity. Our findings provide information about the production of sclerotia and the composition of sclerotial exudate that may be useful to develop strategies to control this disease.

## Introduction

*Panax ginseng* C. A. Meyer is one of most famous perennial medicinal herbs, and it has been used for thousands of years in China (Han et al., 2011). More than 70% of the world’s ginseng production is in China (Wang et al., 2017), and so the control of ginseng diseases in China is extremely important. As a major root disease of ginseng, *Sclerotinia ginseng* has already caused a serious epidemic in northeast China, with incidences ranging from 10% to 15%, and up to 20% in severe cases (Wang et al., 2017). *S. ginseng* is similar to *Sclerotinia sclerotiorum* and has the characteristics of a necrotrophic and soil-borne pathogen (Adams & Ayers, 1979). During the early onset of the disease, there is no obvious difference between healthy and infected plants. As the disease develops, aerial parts of plants show wilting, leaf browning, and softening of the stem base. The root of ginseng, as the main medicinal part, will be soft and rot-like after being infected by *S. ginseng*, causing serious economic losses (Zhou & Fu, 2016). *S*. *ginseng* rarely produces apothecia, and so hyphae are the main route of infection (Wang, Wang & Wu, 1992). After nutrients in the host are depleted, vegetative growth ceases and aerial hyphae aggregate on the surface of ginseng. These events mark the initiation of sclerotia formation (Ordóñez Valencia et al., 2015). The sclerotia remain in the soil or on the diseased plants and serve as the source of infection in the following year.

Sclerotia development consists of three distinct and intertwined stages; initiation, development, and maturation (Rollins & Dickman, 1998; Erental, Dickman & Yarden, 2008). During sclerotia development, exudation of droplets is a common feature (Colotelo, 1973; Cooke, 1969; Liang, Strelkov & Kav, 2010). Studies on such exudates have mainly focused on members of the Ascomycota (Colotelo, 1973; Cooke, 1969; Liang, Strelkov & Kav, 2010) and Basidiomycota (Zarani & Christias, 1997; Pandey et al., 2007; Singh et al., 2002; Aliferis & Jabaji, 2010). Most exudates produced by fungi are produced on aerial hyphae (Aliferis & Jabaji, 2010) and sclerotia (Liang, Strelkov & Kav, 2010). Nutritional conditions, especially the C:N ratio, are critical factors affecting exudate formation in *Metarhizium anisopliae* (Stefan et al., 2010). Carbon sources such as glucose, ribose, galactose, xylose, cellobiose, sucrose, sorbose, and mannitol are often supplied alone or in combination to pathogens to study the relationship between morphogenesis and nutritional factors (Kanwal & Reddy, 2012; Budge & Whipps, 1991; Kuës, 2000). Previous studies have shown that various carbon and nitrogen sources result in differently sized sclerotia in *Morchella* spp. (Kanwal & Reddy, 2012). Similarly, the number, fresh weight, and mean weight of sclerotia produced by an isolate of *S*. *sclerotiorum* were all affected by the sucrose concentration (Budge & Whipps, 1991). Sclerotial development is also affected by non-nutritional factors including light, temperature, pH, humidity, the accumulation of organic acids, phenolic compounds, and other metabolites, polyphenol oxidase activity, contact with mechanical barriers, -SH group modifiers, and osmotic potential (Rollins & Dickman, 1998). In *Pyronema domesticum*, light promoted apothecia formation, while darkness promoted sclerotia formation (Moore-Landecker, 1987). In addition to external factors, some internal factors also affect exudate formation. Dehydration and thickening of cell walls, polymerization of soluble compounds, and decreased moisture content in tissues can all lead to the condensation of exudate droplets on the surface of sclerotia (Daly, Knoche & Wiese, 1967; Willetts & Bullock, 1992; Chet & Henis, 1975; Willetts, 1971).

There is no detailed evidence for the function of exudates, and their roles in diverse niches are unclear. One theory is that exudates help to maintain the internal physiological balance of sclerotia through a selective mechanism to release excess soluble carbohydrates (Cooke, 1969). During the continuous exudation of droplets, some components could be reabsorbed back into the sclerotia to promote further development (Colotelo, 1978). Exudates may also contribute to the role of the sclerotium as an overwintering structure for long-term survival (Adams & Ayers, 1979; Kwon et al., 2014) because of the antifungal, insecticidal, phytotoxic, and antioxidant activities of its components (Aliferis & Jabaji, 2010; Stefan et al., 2010). Pathogenicity is another proposed property of exudates (Aliferis & Jabaji, 2010), and some protein components may be involved in pathogenicity and virulence (Liang, Strelkov & Kav, 2010; Wang et al., 2017). All of the above functions of exudates are yet to be verified.

Many chemical compounds in exudates have been identified using several experimental methods. Aliferis & Jabaji (2010) used three methods to identify more than 90 compounds in the exudate of *Rhizoctonia solani*. Its components included phenolic compounds, carboxylic acids, carbohydrates, fatty acids, and amino acids. Phenolic acids are the major component of exudates, and have been widely studied (Colotelo, 1973; Pandey et al., 2007; Aliferis & Jabaji, 2010; Moore, 1991; Colotelo, 1971). The degree of oxidation of phenolic compounds can affect the color of exudates (Willetts & Bullock, 1992). Carbohydrates are also commonly occurring metabolites released into the culture medium by *R*. *solani* (Vidhyasekaran et al., 1997; Brooks, 2007). Analyses of the ethyl acetate fraction of the sclerotial exudate of *Sclerotinia rolfsii* by high performance liquid chromatography (HPLC) showed that it contained oxalic acid (Singh et al., 2002). Sclerotial exudates of 6-day-old cultures of *S*. *sclerotiorum* were found to contain various salts, cations, lipids, ammonia, amino acids, proteins, and enzymes (Colotelo, 1971).

To the best of our knowledge, no previous study has focused on the exudate produced by *S*. *ginseng*. This is the first study to investigate the effects of different media on the production of exudate by *S*. *ginseng* and to screen for the optimal medium. By integrating different methods, we have identified four types of metabolic substances in the exudate of *S*. *ginseng.* Although this study is still fragmentary and not systematic, our results provide new information about the exudate of *S*. *ginseng*. Understanding the composition of the exudate will help to ascertain its function. These findings lay the foundation for further research on the metabolic mechanisms of fungal sclerotia.

## Material and Methods

### Screening of culture media and collection of exudates

The *S*. *ginseng* strain GSgF-01 was isolated from infected ginseng in Dasuhe Village, Qingyuan County, Liaoning Province, China. This strain was cultivated on 10 different media, as follows: potato dextrose agar (PDA), oatmeal agar (OA), oatmeal-yeast extract agar (OA-YE), 5% malt extract agar (MEA), malt extract-peptone-dextrose agar (MEA-peptone), carrot decoction agar (CA), Sabouraud’s agar (SDA), Richard’s medium agar, V8 juice agar (V8), and Czapek-Dox solution agar (Czapek) at optimal temperature (20 ± 1 °C) in the dark in Petri dishes (diameter, 90 mm). Subculturing was conducted at 5-day intervals. For subculturing, a 5-mm plug was subcultured for another round of the life cycle until the isolate formed sclerotia. When the sclerotia produced the exudate, the liquid was collected from the surface of the sclerotia with a disposable blood collection tube (20 µL) and stored in a 1.5 mL microcentrifuge tube (GEB, Torrance, CA, USA) at −20 °C. To clarify the effects of different media on the growth of *S*. *ginseng* and exudate formation, the following parameters were measured: mycelial growth rate (mm/day), initial formation time of exudate droplets, total quantity of exudate, number of sclerotia per dish, and sclerotial fresh/dry weight. The experiment was repeated twice with three replicates. Statistical significance was evaluated by ANOVA (SPSS 13.0) and the significance level was set at 0.05 (*p* < 0.05). All values are expressed as mean ± S.D.

### Flame atomic absorption spectrometry (FAAS) analysis of sclerotial exudate

Exudate samples (1 mL) were passed through a 0.22 µm Millipore filter and then analyzed to determine cations contents. The parameters for operating the equipment were those recommended by the manufacturer. The sodium (Na), potassium (K), and magnesium (Mg) concentrations in the exudate were investigated by FAAS (Thermo ice 3500, USA) in an oxidizing air-acetylene flame. The absorption of each cation was measured at a specific wavelength. Standard curves were prepared using standard solutions of ions with concentration gradients of 0.0, 0.5, 1.0, 1.5, and 2.0 mg/L, and were fitted by quadratic equations. Blank samples were used to evaluate the presence of compounds not related to the biological material. The ion concentration (*X*) in each sample was calculated using Eq. (1) as follows:

(1) \begin{eqnarray*}X \left( mg/L \right) = \frac{V\times \left( {a}_{1}-{a}_{0} \right) }{{V}_{0}} \end{eqnarray*}

where *V*, *V*_0_, *a*_1_, and *a*_0_ are the total volume of dissolved samples, sample volume, ion content in the sample solution, and ion content in the blank solution, respectively.

### Nessler’s reagent spectrophotometry analysis of sclerotial exudate

The ammonia nitrogen content in each sample was determined by comparison with a standard curve (Lin et al., 2014). A stock solution of ammonia nitrogen was prepared with ammonium chloride (Guaranteed reagent; INESA, Shanghai, China). Then, 0.0, 1.0, 2.0, 4.0, 8.0, 10.0 mL ammonia nitrogen was added to cuvettes, and water was added to complete the volume to 50 mL. Seignette salt (1.0 mL) and 1.5 mL Nessler’s reagent were added to each cuvette and absorbance was detected at 420 nm using a 721-visible spectrophotometer (INESA). The values were used to construct a standard curve, which was used to calculate the ammonia concentration in each sample.

### High performance liquid chromatography (HPLC) analysis of sclerotial exudate

According to the previous study on the composition of the exudate droplets of *Sclerotinia*, eight related organic acids were selected as reference. The reference compounds were oxalic acid (o2si, Charleston, SC, USA), gallic acid, ferulic acid, vanillic acid, chlorogenic acid, tannic acid (ANPEL, Shanghai, China), cinnamic acid (CNW, Duesseldorf, Germany), and caffeic acid (Dr. Ehrenstorfer GmbH, Augsburg, Germany). The purity of these ingredients was >98% as determined by HPLC. For the oxalic acid component of the sample, the chromatographic system was controlled by an Agilent 1260 series HPLC chromatograph equipped with an Athena C_18_-WP column (2.1 × 150 mm, 3 µm). After diluting the sample 10 times, 5 µL sample was injected. The sample was separated using 0.1% phosphoric acid as the mobile phase (flow rate, 1.0 mL/min) for 15 min at a column temperature of 35 °C. The wavelength of the UV–visible detector was set at 210 nm. To detect the other seven compounds, the sample was passed through an Agilent 1260 series HPLC chromatograph equipped with an Innoval ODS 2 column (4.6 × 150 mm, 5 µm). Separation was carried out under the following chromatographic conditions: injection volume, 10 µL; column temperature, 35 °C; UV detection wavelengths, 265 nm and 323 nm; mobile phase, 0.1% phosphoric acid and acetonitrile with gradient elution at a flow rate of 1.0 mL/min.

### Gas chromatography-mass spectrometry (GC-MS) analysis of sclerotial exudate

The samples were vacuum-dried with an appropriate amount of methanol. This process was repeated several times until no water was left in the samples. Next, 1 mL TMS-HT [HMDS (hexamethyl disilazane) and TMCS (chlorotrimethylsilane) in anhydrous pyridine] derivative solution was added to the samples, and then 1 mL *n*-hexane was added to the derivatization system. The *n*-hexane layer was obtained after centrifuging. The four standard substances (mannose, Dr. Ehrenstorfer GmbH; inositol and glucose, ANPEL; trehalose; J&K Scientific, Beijing, China) were prepared in the same way. The sample injection volume was 1 µL. The GC-MS system consisted of an Agilent 7890 instrument (Agilent Technologies, Palo Alto, CA, USA) coupled with an Agilent DB-5 column (30 m × 0.25 mm × 0.25 µm; Agilent Technologies) The temperature program conditions were optimized for analysis of target compounds: the initial temperature of 100 °C (held for 10 min) was raised to 250 °C at 15 °C min^−1^, followed by an isothermal hold for 10 min.

## Results

**Figure 1:**
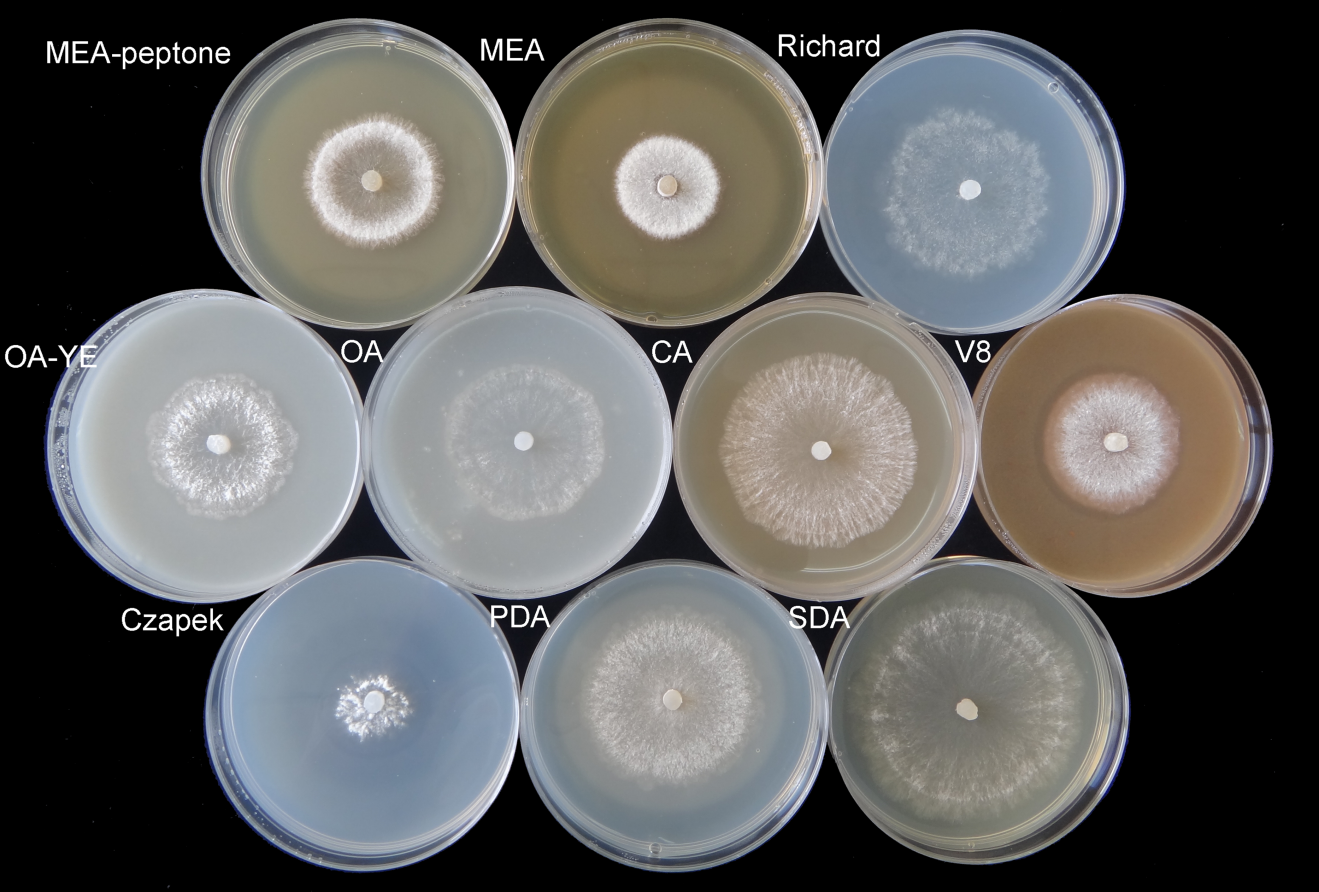
Morphological characteristics of mycelium vegetative growth of *S. ginseng* on 10 different cultivation media after incubation at 20 °C for 4 days. Morphological characteristics of mycelium vegetative growth of *S. ginseng* on 10 cultivation media including: cultivated on potato dextrose agar (PDA), oatmeal agar (OA), oatmeal-yeast extract agar (OA-YE), 5% malt extract agar (MEA), malt extract-peptone-dextrose agar (MEA-peptone), carrot decoction agar (CA), sabourauds agar (SDA), Richard, V8 juice agar (V8) and Czapek-Dox solution agar (Czapek).

Colonies produced a great deal white aerial mycelia on the different culture media at 20 °C in the dark (Fig. 1). On V8, OA, OA-YE, and Richard’s media, some mycelia in the colony center or near the colony margin were light brown, especially those submerged in the medium. The fastest mycelial growth rate of isolate GSgF-01 was on SDA medium (13.47 ± 0.93 mm d^−1^), followed by PDA medium (10.81 ± 1.68 mm d^−1^), and the slowest growth rate was on Czapek medium (4.48 ± 1.73 mm d^−1^) (Table 1). On some media (MEA, MEA-peptone, V8, and Czapek), the slower growth rate was associated with denser hyphae distribution and the formation of distinct white mycelial colonies. With the vegetative growth of mycelia, a clear annular halo formed around aerial mycelia on MEA, MEA-peptone, and Czapek media (Fig. 1). By 11 days, all media except for Richard’s and Czapek were fully covered with mycelia.

**Table 1:** Physiological indicators of sclerotia and exudate droplets on different media. The table contains several information of *Sclerotinia ginseng* on the mycelial growth rate, the number of sclerotia, the fresh weight of sclerotium, the dry weight of sclerotium, the initial formation time of droplets, and the total quality of droplets in different culture media.

Culture medium^a^	Mycelial growth rate (mm d^−1^)^b^	Sclerotia			Exudate droplets	
		Number^c^	Fresh weight (g)^d^	Dry weight (g)^e^	Formation time (d)^f^	Total quality (g)^g^
PDA	10.81 ± 1.68^b^^,^^*^	188 ± 6.66^c^^,^^d^^,^^*^	0.5442 ± 0.0244^d^^,^^*^	0.5073 ± 0.0303^b^^,^^*^	10	1.3108 ± 0.1472 ^a^^,^^*^
OA	8.91 ± 2.19^d^^,^^e^	51 ± 6.11^f^	0.2663 ± 0.0186^e^^,^^f^	0.2263 ± 0.0202^e^	7	0.1983 ± 0.0418 ^d^
OA-YE	8.21 ± 3.44^e^	147 ± 9.29 ^e^	0.2304 ± 0.0145^f^^,^^g^	0.2151 ± 0.0153^e^	6	0.7557 ± 0.0702 ^c^
MEA-peptone	8.10 ± 3.19 ^e^	242 ± 5.03 ^b^	0.7241 ± 0.0271^b^	0.4332 ± 0.0193^c^	7	0.3064 ± 0.1179^d^
MEA	5.98 ± 0.72^f^	203 ± 9.07^c^	0.8683 ± 0.0469^a^	0.6004 ± 0.0066^a^	7	0.5037 ± 0.1360^c^
CA	10.57 ± 2.74^b^^,^^c^	174 ± 6.81^d^	0.2881 ± 0.0134^e^	0.2711 ± 0.0108^d^	7	0.9072 ± 0.1647^b^
SDA	13.47 ± 0.93^a^	57 ± 18.00^f^	0.2272 ± 0.0094^g^	0.1621 ± 0.0084^f^	–	trace^e^
Czapek	4.48 ± 1.73^g^	64 ± 4.16^f^	0.2442 ± 0.0137^e^^,^^f^^,^^g^	0.2202 ± 0.0083^e^	–	trace ^e^
Richard	9.49 ± 1.04 ^c^^,^^d^	62 ± 8.08 ^f^	0.6531 ± 0.0178 ^c^	0.5191 ± 0.0162 ^b^	–	trace^e^
V8	8.27 ± 5.77 ^e^	267 ± 7.00 ^a^	0.1441 ± 0.0167 ^h^	0.1353 ± 0.0188 ^g^	6	0.6091 ± 0.1068 ^c^

**Notes.**

* Means followed by the same letter within each column are not significantly different (*P* > 0.05) according to least significant difference (LSD) test.

– Means no exudation of droplets was observed in that three culture media.

a Means mycelial plug was inoculated in different culture medium.

b Means daily mycelial growth rate was measured on different culture medium with standard deviations.

c Means average sclerotial number per dish was counted on different culture medium with standard deviations.

d Means average fresh weight of sclerotia of three dishes was weighed from different culture medium with standard deviations.

e Means average dry weight of sclerotia of three dishes was weighed from different culture medium with standard deviations.

f Means initial formation time of exudate droplets was recorded on different culture medium.

g Means average total quality of exudate droplets of 30 dishes was weighed from different culture medium with standard deviations.

**Figure 2:**
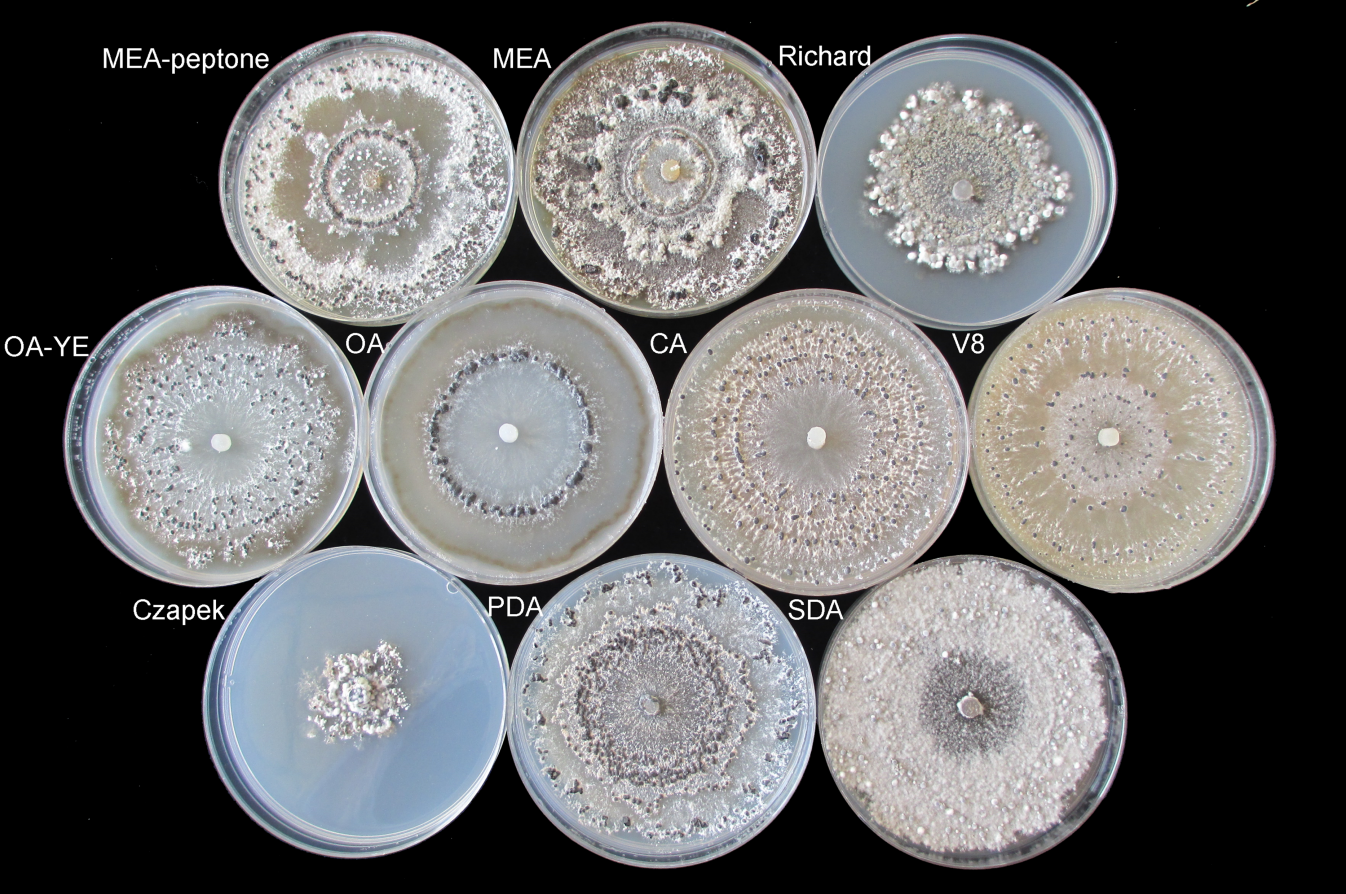
Morphological characteristics of sclerotia of *S. ginseng* on 10 different cultivation media after incubation at 20 °C for 15 days. Morphological characteristics of sclerotia of *S. ginseng* on 10 cultivation media including: cultivated on potato dextrose agar (PDA), oatmeal agar (OA), oatmeal-yeast extract agar (OA-YE), 5% malt extract agar (MEA), malt extract-peptone-dextrose agar (MEA-peptone), carrot decoction agar (CA), sabourauds agar (SDA), Richard, V8 juice agar (V8) and Czapek-Dox solution agar (Czapek).

**Figure 3:**
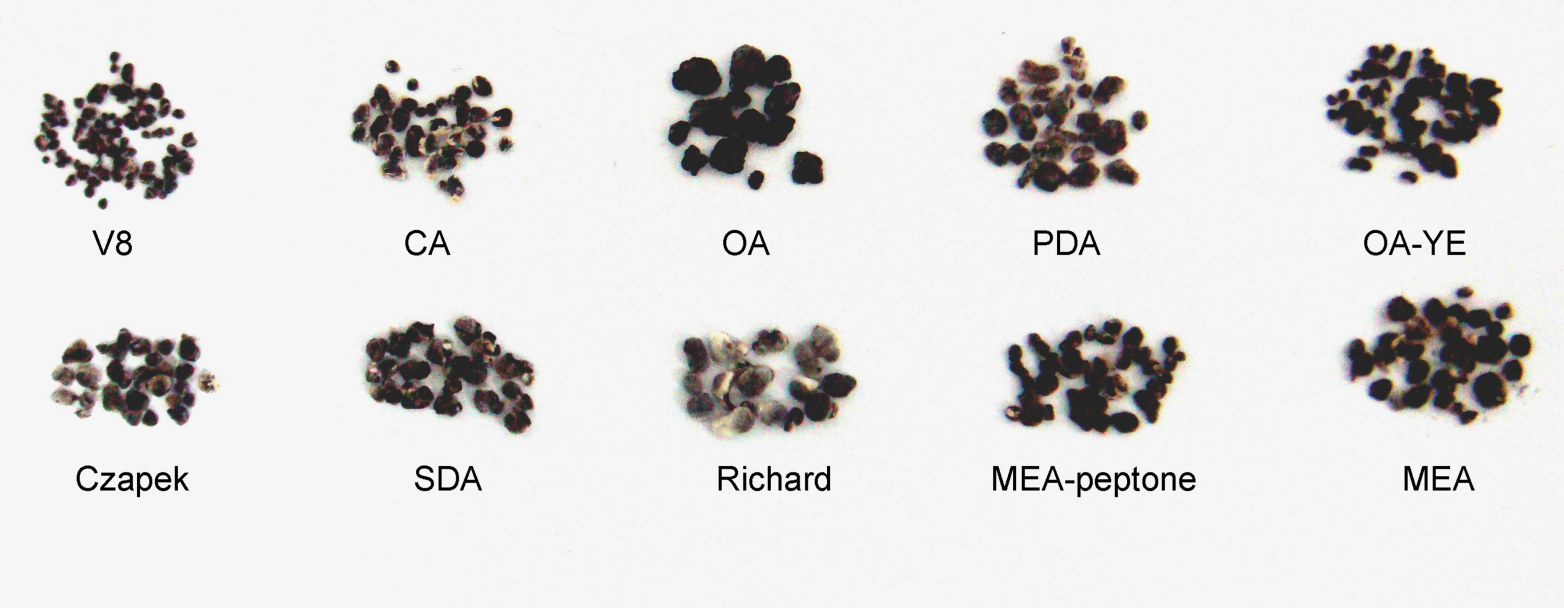
Morphological characteristics of sclerotia of *S. ginseng* derived from 10 different cultivation media. Morphological characteristics of sclerotia of *S. ginseng* derived from 10 different cultivation media including: cultivated on potato dextrose agar (PDA), oatmeal agar (OA), oatmeal-yeast extract agar (OA-YE), 5% malt extract agar (MEA), malt extract-peptone-dextrose agar (MEA-peptone), carrot decoction agar (CA), sabourauds agar (SDA), Richard , V8 juice agar (V8) and Czapek-Dox solution agar (Czapek).

On OA medium, only a single circle composed of sclerotia formed. Sclerotia were firmly attached to the surface of the agar along the edge of mycelial colonies on Richard’s medium, but were generally arranged in concentric rings on the other media (Fig. 2). The morphology of sclerotia varied among the different culture media. The shapes of sclerotia ranged from spherical to elongated. Some sclerotia aggregated to form irregular shapes, which existed alone or contiguously on the agar medium (Figs. 2 and 3). The largest number of sclerotia formed on V8 medium, but they were also the smallest sclerotia. The fewest sclerotia formed on OA medium, but their size was larger than those formed on other media (Fig. 3) (Table 1). The maximum dry/fresh weight of isolate GSgF-01 was on MEA medium (0.6004 ± 0.0066/0.8683 ± 0.0469 g) and the minimum dry/fresh weight was on V8 medium (0.1353 ± 0.0188/0.1441 ± 0.0167 g).

After 6 d, sclerotia with exudate droplets were visible on V8 and OA-YE media. Although the fastest growth rate of vegetative mycelia was on SDA medium, sclerotial development on this medium lagged behind that on other media. Similar to the sclerotia on Czapek and Richard’s media, sclerotia on SDA medium produced only trace amounts of exudate. The amount of exudate was significantly higher on PDA medium (1.3108 ± 0.1472 g) than on other media. Therefore, PDA medium was selected as the optimal medium for producing sclerotial exudate (Table 1).

The contents of K, Na, and Mg ions detected in the exudate were 624.2, 107.3, and 65.4 mg/L, respectively. The free ammonia concentration was 113.86 mg/L, lower than that detected in the exudate produced by a 5-day-old S. sclerotiorum culture (Colotelo, 1973).

**Figure 4:**
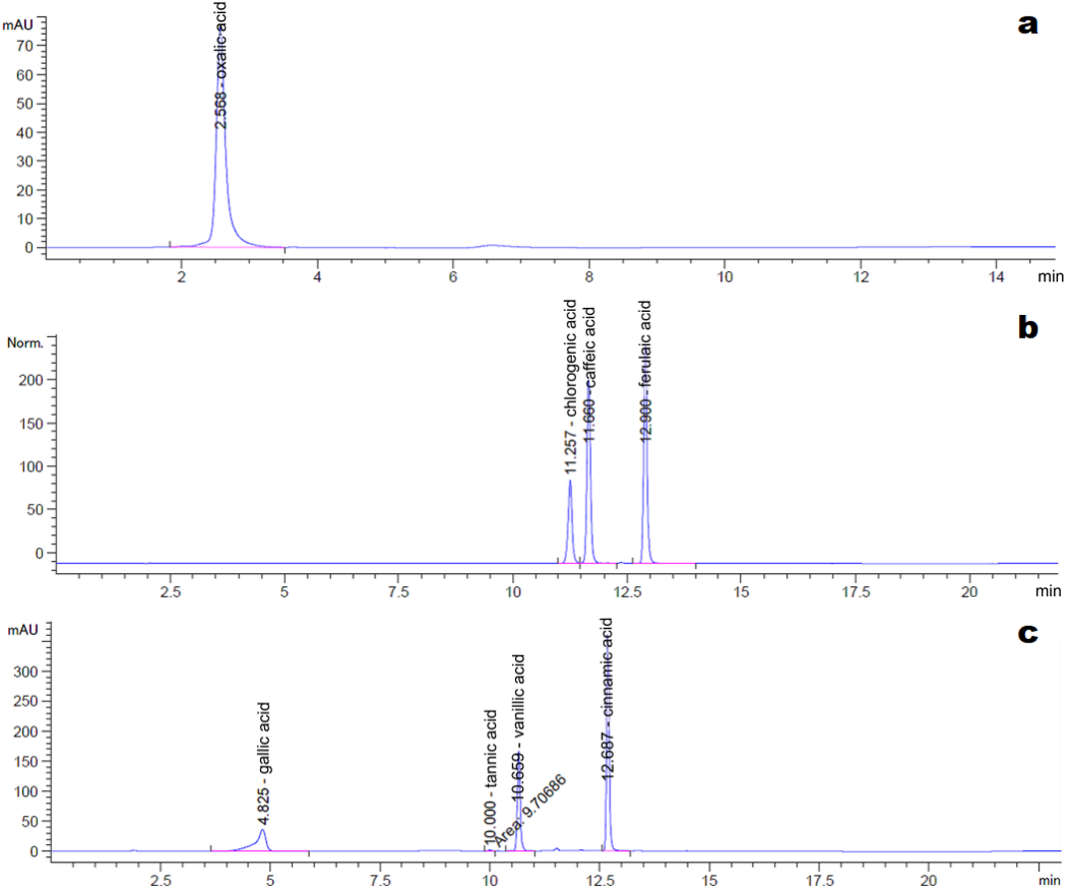
Chromatogram of reference standards. (A) Oxalic acid; (B) Chlorogenic acid, caffeic acid and ferulic acid; (C) Gallic acid, tannin acid, vanillic acid and cinnamic acid.

**Figure 5:**
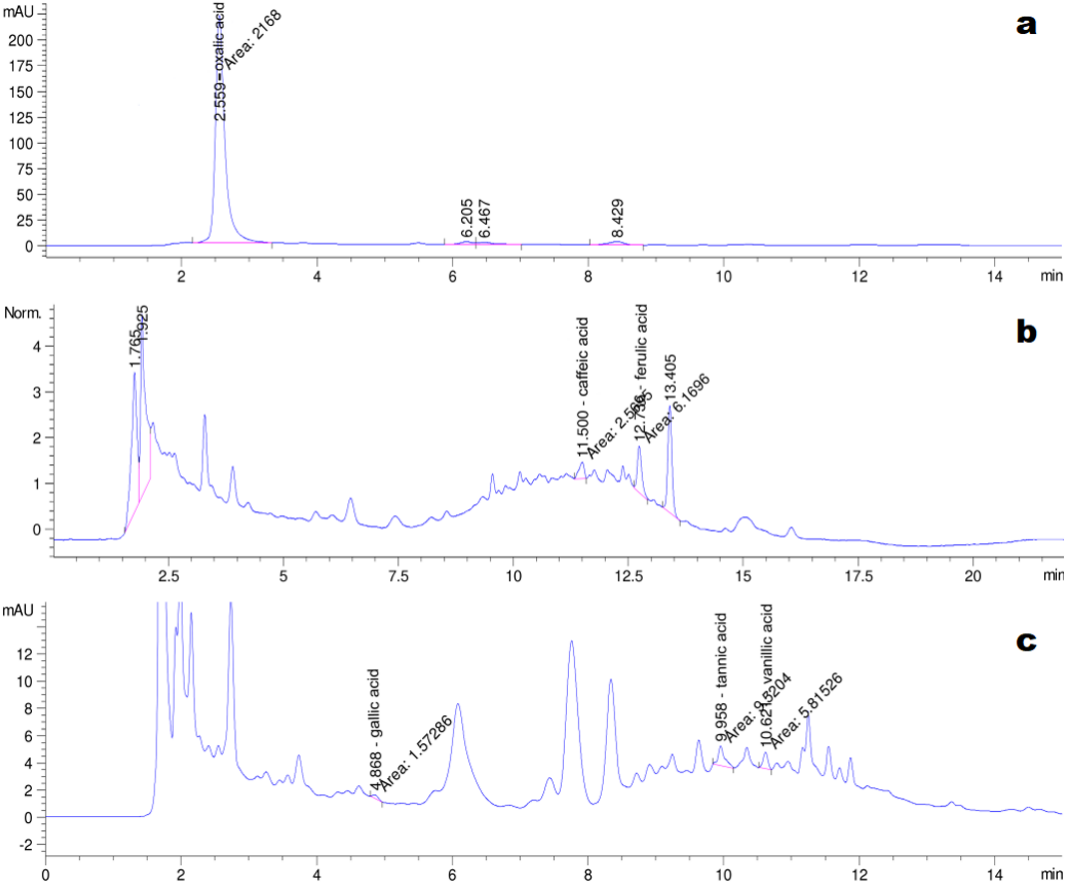
Chromatogram of exudate droplets. (A) Oxalic acid; (B) Caffeic acid and ferulic acid; (C) Gallic acid, tannin acid and vanillic acid.

The HPLC chromatograms of the eight organic acid standards are shown in Fig. 4. Only six of the eight organic acid compounds were detected in the exudate (Fig. 5). The oxalic acid content in the exudate was relatively high. The tannic acid content in the exudate (19.70 mg/L) was much higher than those of other identified phenolic acids. Chlorogenic acid and cinnamic acid were not detected in the exudate of S. ginseng.

**Figure 6:**
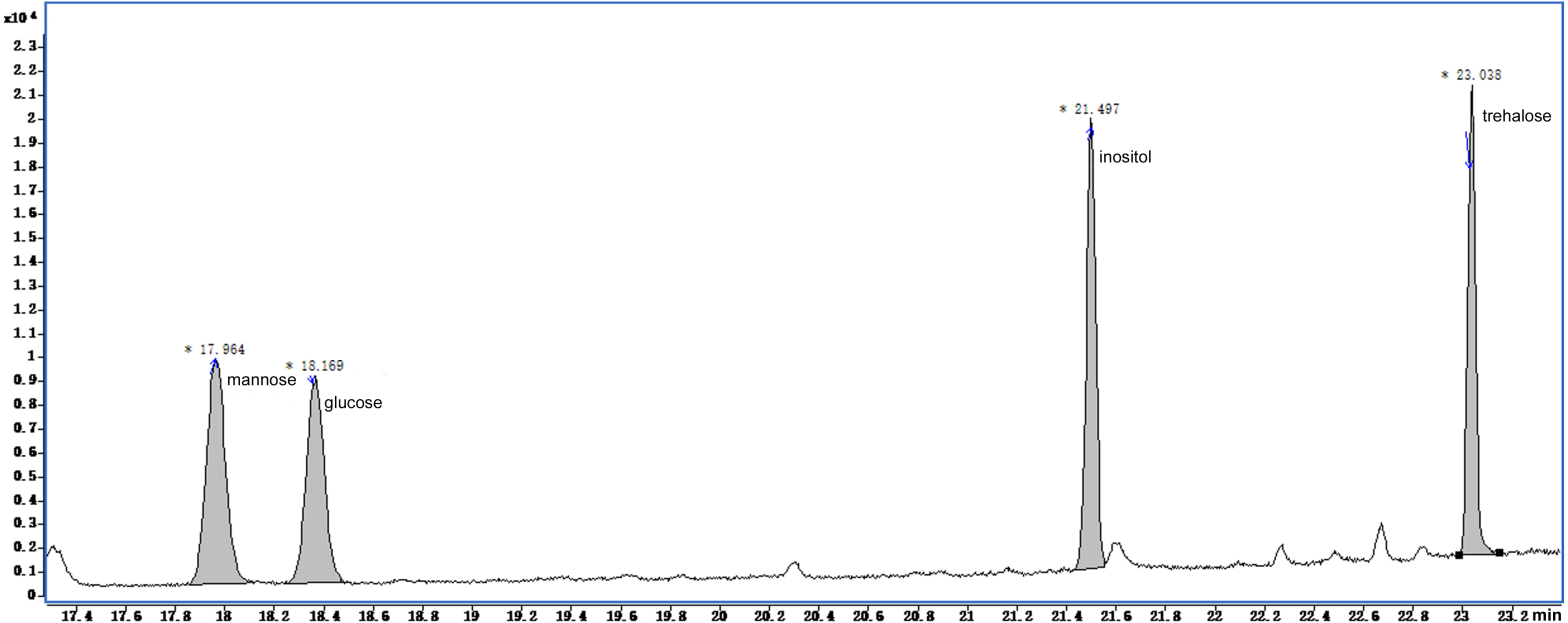
Total ion current chromatogram of silylation derivatives of the 4 standards from soluble sugars. There were four standards substances identified including mannose, glucose, inositol and trehalose.

**Figure 7:**
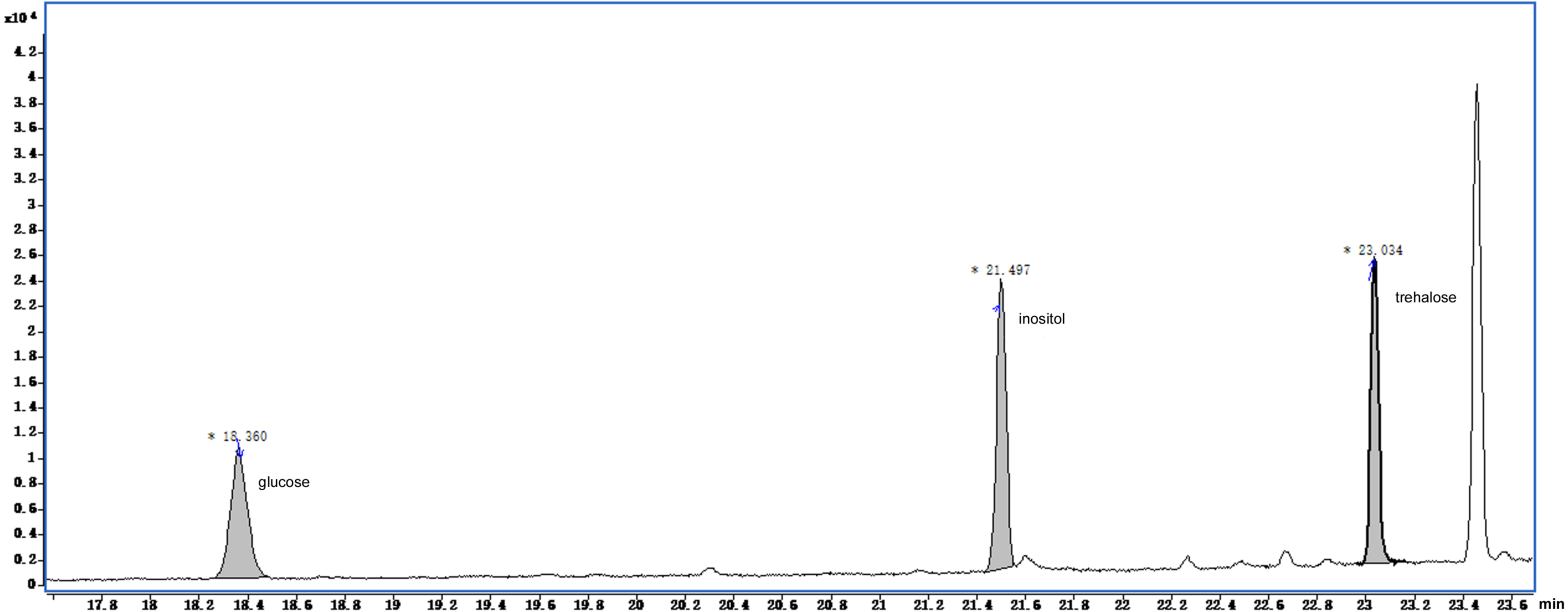
Total ion current chromatogram of silylation derivatives of the exudate droplets. There were three substances identified including glucose, inositol and trehalose.

The GC-MS chromatograms of the silylated derivatives of four soluble sugar standards are shown in Fig. 6. The exudate contained inositol, glucose, and trehalose, but not mannose Fig. 7. Quantitative analyses of the exudate revealed that glucose (6.1 mg/L) was relatively more abundant than other sugars. Besides inositol, glucose, and trehalose, one unidentified peak was detected. The compound represented by this peak was more abundant than the other identified compounds, suggesting that it might play a major role in the function of the exudate.

## Discussion

*S*. *ginseng* is a saprophytic fungus that parasitizes the host and survives in soil for a long time. Fungi in the Sclerotiniaceae have a wide range of hosts and can infect multiple parts of the host including the root, stem, leaf, and seed (Boland & Hall, 1994). Because these fungi are capable of deriving nutrients from a wide range of species and plant tissues, it was available to explore the effects of different culture media on the growth and development of *S*. *ginseng*. We evaluated the mycelial growth rate, total quantity of exudate, number of sclerotia, sclerotial fresh weight and dry weight, and sclerotial distribution of *S. ginseng* grown on 10 different media. We speculated that sclerotial development and metabolism would be affected by different culture conditions. Information about the growth of *S. ginseng* on different media can provide a reliable direction for future studies on sclerotia development and metabolism.

On SDA medium, *S. ginseng* showed the fastest mycelial growth rate, but was slow to produce sclerotia, and formed fewer sclerotia that produced less exudate than *S. ginseng* growing on the other media. Although the slowest mycelial growth rate was on Czapek medium, pigmented sclerotia formed earlier on Czapek medium (6 days) than on other media, and the sclerotia were irregularly distributed around the hyphal plug. These observations suggested that the conditions suitable for mycelial growth were not necessarily suitable for the formation of sclerotia. Thus, the two processes likely have interwoven but separate development mechanisms. The Czapek and Richard media did not become fully covered with mycelia, and unpigmented sclerotia remained on these media for a long time. The outermost layer of mature sclerotia on these media was greyish (Fig. 3).

As shown in the chromatogram, the composition of the exudate was complex. It is important to comprehensively identify its constituents to understand its functions. Comparing the composition of exudates between *S*. *sclerotiorum* (Colotelo, 1971) and *S*. *ginseng*, the K ion content was higher in the *S*. *sclerotiorum* exudate than in the *S*. *ginseng* exudate, whereas the contents Na and Mg ions were much lower (Colotelo, 1971). As reported in previous studies, metal ions at specific concentrations can affect mycelial growth and the formation, dry weight, and size of sclerotia (Moromizato et al., 1991). Dynamic monitoring of ions has also shown that ions are selectively absorbed into the sclerotia (Colotelo, 1973).

Ammonia was detected in the exudate of *S*. *ginseng*, like in the exudate of *S*. *sclerotiorum* (Colotelo, 1973). Ammonia can raise the pH of the immediate environment. Previous studies have noted that pathogen infection is enhanced in an alkaline environment (Vylkova, 2017). During the necrotrophic development of *Colletotrichum gloeosporioides* in tomato, tissue necrosis was found to be associated with rapid ammonia release, which triggered the expression of virulence factors (Shnaiderman et al., 2013). Colotelo (1973) speculated that exudates containing high levels of free ammonia could cause disease symptoms. Thus, it was suggested that the exudate might have a function in pathogenesis. Ammonia is a predominant bacterial volatile organic compound that has been shown to induce trap formation in the nematode-trapping fungus *Arthrobotrys oligospora* (Su et al., 2016).

Oxalic acid is a phytotoxin secreted by many plant pathogenic fungi, and is required for effective pathogenesis (Cessna et al., 2000; Kim, Min & Dickman, 2008; Xu et al., 2015). Oxalic acid reduces pH and is involved in acidity-induced activation of enzymes, chelation of calcium ions, and inhibition of reactive oxygen species. Its toxic effect may enable the fungus to colonize host plants (Cessna et al., 2000; Williams et al., 2011; Walz et al., 2008; Dutton & Evans, 1996). Oxalic acid is also an elicitor of plant programmed cell death during *S*. *sclerotiorum* disease development (Kim, Min & Dickman, 2008). The presence of oxalic acid in the exudate suggested that it may play a role in the pathogenesis of *S*. *ginseng*.

We detected various phenolic acids including gallic acid, ferulic acid, vanillic acid, caffeic acid, and tannic acid in the exudate. Phenolic compounds are involved in oxidation–reduction processes, which are important for, and related to, the synthesis of melanin (Abo Ellil, 1999; Butler, Gardiner & Day, 2005). Melanin may affect the normal development of sclerotia, formation of a hard cell wall, and the overwintering of pathogens. Phenolic acids are also involved in allelopathy in the soil and in plant-induced resistance, and affect the antimicrobial activity and antioxidant activity of the pathogen (Singh et al., 2002; Jain et al., 2015). By inhibiting the germination of spores from other pathogens in the soil, phenolic acids can also protect the pathogen itself against degradation by other microorganisms (Pandey et al., 2007). Aliferis & Jabaji (2010) found that the amount of ferulic acid and its ethyl ester was correlated with antifungal activity, as measured by the inhibition of germination of spores of other fungi. The exudate also affected pathogenicity (Liang, Strelkov & Kav, 2010; Aliferis & Jabaji, 2010), which was related to the release of oxalate into the culture liquid (Singh et al., 2002).

Inositol, glucose, and trehalose were identified in the exudate of *S*. *ginseng,* but mannose was not detected. Trehalose, inositol, and glucose were also detected in the exudates of *S*. *sclerotiorum* and *S*. *trifoliorum*. The exudate of *S*. *sclerotiorum* was also found to contain mannitol (Cooke, 1969). Glucose was the most abundant carbohydrate in the exudate of *S. ginseng,* but was found at only trace levels in the exudates of *S*. *sclerotiorum* and *S*. *trifoliorum* (Cooke, 1969). Although these fungi seemed to produce a similar range of carbohydrate compounds, they showed some differences in the types of carbohydrates they produced, and this may related to the nutrients in the culture media. As described in previous studies, carbohydrates have multiple roles in fungi. They are involved in the regulation of growth and development (Liang, Strelkov & Kav, 2010), the control of differentiation of fruiting bodies (Zhou et al., 2016), and modification of the fungal cell wall (Latgé, 2007). Vidhyasekaran et al. (1997) found that the main components of *R*. *solani* toxin were carbohydrates, including glucose, mannose, N-acetylgalactosamine, and N-acetylglucosamine. Sriram et al. (2000) also reported that a fungal toxin contained a mixture of carbohydrates including α-glucose and mannose.

## Conclusion

There is a close relationship between nutritional conditions and the morphogenesis of pathogens. We screened 10 media, and found that PDA was the optimal medium for exudate production. Using four analytical platforms, we identified oxalic acid, gallic acid, ferulic acid, vanillic acid, caffeic acid, tannin acid, inositol, glucose, trehalose, K, Na, Mg, and ammonia as components of the exudate. Many of these identified components in the sclerotial exudate of *S*. *ginseng* have been reported to play roles in pathogenicity. Information about the conditions that lead to sclerotia formation and the composition of the exudate may shed light on the mechanism of sclerotial formation and the role of the exudate in pathogenicity.

##  Supplemental Information

10.7717/peerj.6009/supp-1 Supplemental Information 1 Supplement dataset of Table 1 The excel worksheet contains several information of *Sclerotinia ginseng* on the mycelial growth rate, the number of sclerotia, the fresh weight of sclerotium, the dry weight of sclerotium, the initial formation time of droplets, and the total quality of droplets in different culture media.

## Additional Information and Declarations

### Data Availability

The following information was supplied regarding data availability: Raw data are available as Supplemental Files.
